# Penalty of Overwork

**Published:** 1861-12

**Authors:** 


### PENALTY OF OVERWORK.

Overwork, and its penalty, worn out brain and early death, are common enough amongst ourselves, but perhaps there is no better example of the beginning, middle, and end of such a case than is afforded by the late Augustus Welby Pugin, a celebrated English architect, and narrated in Mr. Ferry's very interesting "Recollections of Pugin." He was "delicate as an infant;" when at school "he showed remarkable aptitude for acquiring knowledge," and whether "in Greek, Latin, mathematics, or any other branch of education, would learn in twenty-four hours what it took other boys many weeks to acquire;" he was quick, fluent in speech, voluble, dogmatic, and vehement in manner; he hated children's society, and loved that of his elders. From his precocity of talent, he was largely employed as an artist when a mere stripling, and was designing George the Fourth's furniture at Windsor when sixteen. He entered into business, set up an establishment of artist workmen, got into pecuniary difficulties, and was arrested when eighteen. He married; and was husband, father, and widower before he had completed his twentieth year. Then followed twenty years of the most intense excitement and exhausting labor; rapid journeys; a second and third marriage, and two, at least, most bitter disappointments in love between them; a change of religion, and a perfect tempest of controversial pamphleteering. Habits of life, of rapid and restless locomotion, and of severe labor when at home; rising at six; two hours' hard work before breakfast, "which seldom lasted more than *seven minutes*;" hard work again till dinner, "when his fare was of the simplest description, without wine or malt liquor, and this meal generally lasted *a quarter of an hour*;" after dinner more work till bed time; "when he returned from town he always wrote letters in the railway carriage." What wonder that at the age of forty he became irritable and nervous, and quarrelled with his oldest friends; or, as he described himself, "thin, trembling, hollow-eyed, changed, and yet working tremendously at times. Soon after, meeting his Medical attendant at Ramsgate, where he lived, he told him a story of the foundering of five merchant ships, which was sheer hallucination; this was followed speedily by complete mental decay and early death, just at the age when experience begins to ripen, and a man's early work ought to bear fruit for himself, his family, and the world. Pugin is a man whose influence is seen and felt in every street and every house, in the substitution of the bold, picturesque, Gothic outlines, and harmoniously colored materials of modern buildings, for the monotonous pseudo-classic of the last generation; in the graceful forms of domestic furniture, and the truer and more natural school of art. If he had worked less, taken more time to his meals, and quieted his brain with tobacco or wine, so as not to live so fast, he might, perhaps, have been living now. —*London Medical Times.*

